# Is assessing trunk muscle endurance in military with sub-acute and chronic low back pain clinically meaningful?

**DOI:** 10.3389/fspor.2023.1173403

**Published:** 2023-05-11

**Authors:** Benoit Pairot de Fontenay, Marc Perron, Chantale Gendron, Pierre Langevin, Jean-Sébastien Roy

**Affiliations:** ^1^ University of Lyon - University Claude Bernard Lyon 1, EA 7424 - Inter-University Laboratory of Human Movement Science, Villeurbanne, France; ^2^ Ramsay Santé, Clinique de la Sauvegarde, Lyon, France; ^3^ Department of Rehabilitation, Faculty of Medicine, Laval University, Quebec City, QC, Canada; ^4^ Canadian Forces Health Services Group, Valcartier Garison, Quebec City, QC, Canada; ^5^ Physiothérapie Interactive, Quebec City, QC, Canada; ^6^ Centre for Interdisciplinary Research in Rehabilitation and Social Integration, Quebec City, QC, Canada

**Keywords:** rehabilitation, strength, clinical evaluation, metrology, endurance

## Abstract

**Introduction:**

Trunk muscle endurance (TME) tests are commonly used by clinicians to assess muscle performance changes in response to rehabilitation in patients with low back pain (LBP). The aim of this study was to assess the responsiveness of three TME-tests in patients with LBP and to evaluate the relationships between changes in TME and improvement in self-reported function.

**Materials and Methods:**

Eighty-four LBP patients were evaluated at baseline and after completion of a 6-week training program. Function was assessed with the modified Oswestry Disability Index (ODI) while TME was estimated using three tests: (1) the Biering-Sørensen, (2) the side bridge endurance tests (both sides), and (3) the trunk flexor endurance test. The standardized response mean (SRM) and the minimal clinical important difference (MCID) for each TME-test, and the relationships between changes in TME and improvement in ODI were calculated.

**Results:**

SRMs were small to large for TME-tests (range: 0.43–0.82), and large for the ODI (2.85) and no clinically useful MCID was identified for the TME-tests (area under the curve below 0.70). No significant correlations were found between changes in the TME and change in ODI scores (*r* < 0.15; all *P* > 0.05).

**Conclusion:**

Our results show a weak responsiveness of TME-tests in patients with LBP. There was no association between endurance performance change and self-reported functional change. TME-tests may not be a key component of rehabilitation monitoring in patients with LBP.

## 1. Introduction

The most recent clinical practice guidelines and systematic reviews on primary care management of patients with non-specific low back pain (LBP) unanimously recommend exercise therapy to reduce pain and disabilities (1–5). Although current evidence does not make definitive recommendations on which type of exercise is most beneficial, emerging evidence suggests that strength or endurance training of trunk muscle groups and coordination or core stabilization exercises are more effective compared to cardiorespiratory or stretching training (6).

Muscle strength and endurance are two characteristics of muscle performance that are targeted by exercise therapy in patients with non-specific LBP (6). It is well accepted that low back pain is not a homogeneous condition (7, 8). However, improvement of the lumbopelvic-hip complex muscle performance following exercises led to decreased pain intensity and improved function in a heterogeneous cohort of patients with non-specific LBP (9). Considering that an improvement in the performance of trunk muscles likely contributes to the reduction of pain and disability, clinicians should be concerned about measuring muscle strength or endurance with valid tests to establish baseline deficits, track changes over time and confirm the benefits of their interventions in LBP patients. In clinical settings, health professionals frequently use inexpensive and simple endurance tests, such as holding time tests. These tests are designed to assess trunk muscle endurance and have be shown to be valid for such assessments (10). They include the Biering-Sørensen test for back extensor muscles (11), as well as the side bridge test for external / internal abdominal obliques and transvs. abdominis, and trunk flexor endurance tests for rectus and transvs. abdominis (12). Sustaining low intensity muscle contraction during long duration is likely to reflect core stability (13). Moreover, their reliability is moderate to excellent (intraclass correlation coefficient: 0.77–0.97) (12, 14, 15) and reference test values have been published for healthy adolescents and adults (14, 16, 17). However, the responsiveness of these tests is unknown.

Responsiveness can be assessed with different metrics, such as the standardized response mean (SRM) which reflects the standardized change over time relative to the between patient change variability (i.e., mean change divided by standard deviations of the change) (18), and the minimal clinical important difference (MCID) which represents a clinically significant change for the individual patient. Furthermore, the relationship between the amount of change in holding time of these tests and the reduction in pain and disability needs to be established to justify their use as follow-up measures in patients with LBP.

The primary objective of the current study was to assess the responsiveness of three trunk muscle endurance tests: (1) the Biering-Sørensen, (2) the side bridge endurance tests (both sides), and (3) the trunk flexor endurance test. More specifically, we aimed to calculate the SRM and determine the MCID for each of these trunk muscle endurance tests. The second objective was to investigate the longitudinal validity of endurance tests by looking at the relationships between changes in endurance time of these three trunk muscle endurance tests and change in self-reported function in patients with LBP.

## 2. Methods

This is a secondary analysis from a hypothesis-driven study designed to create a clinical prediction rule for a favorable outcome following a multi-station full-body supervised exercise program that has previously been published (19).

### 2.1. Participants

Military members (support duties [office, supply: physical activity participation ≥2*/week] and operational duties [infantry, artillery: physical activity participation ≥5*/week]) consulting at Valcartier Health Centre for LBP were consecutively recruited. Inclusion criteria were: (1) to be aged 18 and older and present with an episode of subacute or chronic non-specific LBP with or without radiation to the lower limbs (20, 21), and (2) to have a minimum score of 17/100 on the Modified Oswestry Disability Index (ODI) at the initial evaluation [given that the smallest detectable change of the ODI is 17/100 (22)]. Non-specific LBP is defined as LBP that cannot be attributed to a specific condition (e.g., vertebral fracture, spinal or foraminal stenosis, infections) (1).

Exclusion criteria were: (1) previous surgery to the spinal column, lumbar spine injection in the past six weeks, signs of upper motor neuron lesions (bilateral paresthesia, hyperreflexia or spasticity), serious medical conditions or specific LBP (e.g., tumours, fracture, rheumatoid arthritis, osteoporosis, spinal or foraminal stenosis) and unavailability to participate in the 6-week exercise program, and (2) acute LBP signs and symptoms (e.g., onset of constant and intense pain [>5/10] < 7 days, severely limited lumbar range of motion [more than 50% in at least 2 directions], obvious lateral shift). In the latter case, the inclusion was postponed until the patient condition had improved and the participation in the study was deemed safe.

Participation in the study was voluntary and an informed consent form was signed by all subjects before enrollment. This study was approved by the local ethics committee (CIUSSS de la Capitale-Nationale; # 2013-302).

### 2.2. Study design

This was a single arm prospective study in which, following the baseline evaluation, participants took part in a 6-week exercise program. They were reassessed at the end of the program. At baseline, participants filled out a sociodemographic questionnaire and the ODI. Thereafter, a physiotherapist assessed the endurance of the trunk muscles. The ODI and muscle endurance tests were reassessed at the end of the 6-week exercise program and participants rated their global perception of change following the intervention with a Global rating of change (GRC) scale (23).

### 2.3. Outcome measures

The baseline and final evaluations were carried out by four experienced physiotherapists who completed a 2-hour training session in order to standardize the evaluation protocol.

#### 2.3.1. Modified oswestry disability index

The modified Oswestry Disability Index (ODI) is a self-administered questionnaire consisting of 10 items that assess the interference of LBP with activities of daily living. The final score ranges from 0 to 100. Zero represents no disability and 100 represents maximum disability possible. The ODI has excellent reliability (intraclass correlation coefficient: 0.88, 95% CI: 0.77–0.94) (24).

#### 2.3.2. Endurance tests of the trunk muscles

Endurance tests of the trunk muscles measure the maximum time, in seconds, that the participant can maintain a static test position. Three tests, in the following order for all participants, were assessed to determine trunk muscle endurance performance: *Biering-Sørensen test, side bridge test* (performed on both sides) and *trunk flexors endurance test*. Two minutes of rest were applied between each test to limit muscle fatigue. Descriptions of the characteristics of each test are provided in Supplementary material.

#### 2.3.3. Global perception of change

The global perception of change after the intervention was assessed with a GRC scale (23). The participants were first asked if their condition was deteriorated, unchanged or improved following participation in the program. Those who were deteriorated or improved were asked to rate the magnitude of that change on a seven-point scale (1–7) (Table 1).

**Table 1:** Associations between changes in the trunk muscle endurance tests and the change in the modified Oswestry disability index (ODI) scores after completion of the 6-week training program.

	ODI
Right-side bridge test	*r* = 0.150
Left-side bridge test	*r* = 0.104
Trunk flexor endurance test	*r* = 0.086
Biering-Sørensen test	*r* = 0.106

r: Pearson's correlation coefficient.

### 2.4. Intervention

The participants took part in a 6-week multi-station full-body supervised exercise program aimed to improve core stability and strength, load tolerance, and trunk endurance. This program is described in detail in Supplementary material S1. The program was administered two to three times per week. The duration of each session was individualized between 45 and 60 min. The supervising physiotherapist stopped the session when the fatigue led to the deterioration of the quality of movements. It was composed of 7 thematic stations: (1) hip strengthening and control; (2) squat and its variants; (3) elastic bands and the Bodyblade; (4) abdominal planks and their variants; (5) abdominal strengthening; (6) back extensor strengthening; and (7) lifting techniques. Muscle strength and endurance as well as neuromuscular control were targeted in each thematic station to improve core stability, strength and endurance of trunk muscle with a task-oriented approach. For each station, a progression of exercises was determined to increase the demand on trunk stabilizer muscles (increased load, time of contraction and/or complexity of the task). The objective was to prompt maximal effort while maintaining proper movement quality before increasing the level of difficulty. To improve endurance of trunk muscles, participants made 15–25 repetitions or kept static positions for 15–30 s with rest periods corresponding to the time needed to get from one exercise station to another (25). When fatigue led to the deterioration of the quality of movements, the exercises were paused or stopped. The initial choice of exercises was determined by the supervising physiotherapist according to: (1) the severity of the condition (constant or non-constant pain, disturbed sleep, limitation and restriction level according to the ODI results), (2) the most limited plane of motion (as the prescribed exercises were primarily carried out in the planes of motion that showed limited mobility or aberrant movements); and (3) the quality of exercise execution. During the course of the study, no other physiotherapy intervention modalities were performed (e.g., manual therapy).

### 2.5. Statistical analyses

Statistical analyses were conducted using the R software (version R.2.7.2.; R Foundation for Statistical Computing, Vienna, Austria) and Excel (Excel 365; Microsoft Corporation, Redmond, Washington, USA).

#### 2.5.1. Descriptive statistics

The mean and standard deviation for each outcome measure at baseline and the 6-week follow-up were reported. Data normality was assessed and confirmed before performing parametric testing. Paired t-tests were used to determine significant differences between pre- and post-intervention.

#### 2.5.2. Responsiveness

##### 2.5.2.1. Standardized response mean

Responsiveness of each endurance trunk test and of the ODI was estimated using SRM [mean change divided by standard deviation (SD) of change] with confidence intervals at 95% (95% CIs) (26). SRM calculation assumes that all participants change in the same direction, therefore, responders were defined as those who were improved according to the GRC at the 6-week follow-up (GRC ≥ 1). A SRM was considered large if ≥0.8, moderate if between 0.5 and 0.8, and small if between 0.2 and 0.5 (27).

##### 2.5.2.2. Minimal clinically important difference

The MCID is the smallest change that represents a clinically significant change for the individual patient. The GRC was used as anchor to differentiate participants who were moderately to greatly improved (4–7 on GRC) from those who were stable to slightly improved (0–3 on the GRC) at the 6-week follow-up. A receiver-operating characteristic (ROC) curve was constructed for each endurance trunk test. Sensitivity and specificity of the change scores of moderately to greatly improved (4–7 on GRC) and stable to slightly improved (0–3 on the GRC) participants were plotted.

Additionally, the probability that scores correctly discriminate between responders and non-responders (accuracy) is depicted by the area under the curve (AUC). An AUC under 0.7 is considered as insufficient, whereas an AUC of 0.7–0.8 is considered adequate, and an AUC of 0.8–0.9 is considered excellent (28).

#### 2.5.3. Longitudinal validity

As a prerequisite, we checked that there was no effect of age on the change in holding time for each of the three trunk muscle endurance tests and the change in ODI scores. Then, associations between changes in holding time of endurance trunk tests and changes in ODI scores were tested using the Pearson correlation coefficient. Associations were classified as very-low (0.0–0.3), low (0.31–0.5), moderate (0.51–0.7), high (0.71–0.9), or very high (0.9–1.0) (29). An alpha level of 0.05 was used for significance.

## 3. Results

Of the 108 participants initially included, 84 completed the endurance tests at baseline and at the follow-up evaluation (Table 2). The results of 84 of the 672 tests performed by participants (22/168 left side bridge tests; 21/168 right side bridge tests; 34/168 trunk flexor tests; 7/168 Biering-Sørensen tests, respectively) were withdrawn from the analyses because participants had to interrupt the test due to an unbearable pain (located at the low back, upper or lower limb).

**Table 2:** Baseline participants’ characteristics (*n* = 84).

Variables	
Age (years)	37.8 ± 9.2
Gender (% men)	89
Operational duties (%)	26
Support duties (%)	44
Weight (kg)	86.1 ± 18.3
Height (m)	174.2 ± 7.7
History of LBP (%)	74
Number of months since last onset of LBP	26.5 ± 60.7
Number of treatments received before baseline evaluation	5.9 ± 11.0
Referred pain in the lower limb (%)	33
FABQ-work subscale (0–42)	21.29 ± 10.47
FABQ-physical activity subscale (0–24)	13.18 ± 5.06
Medication: non-opioid pain killer / opioid pain killer	35 / 3
Average pain in previous 48 h (0–10)	3.08 ± 1.38

LBP, low back pain; FABQ, Fear avoidance beliefs questionnaire; NSAID, nonsteroidal anti-inflammatory drug.

### 3.1. Descriptive statistics

Mean holding time for the three trunk tests and ODI score at baseline and at the 6-week follow-up are presented in Table 3. Of the 84 participants, none were deteriorated after the intervention, 9 reported no improvement (GRC = 0), 75 reported improvements (GRC ≥ 1) and 55 were at least moderately better (GRC ≥ 4) (Table 3).

**Table 3:** A Baseline and 6-week follow-up holding time for the four trunk endurance tests and ODI scores.

	Baseline (s) Mean ± SD	6-week (s) Mean ± SD	SRM (95% CI)
Right-side bridge test	63.5 ± 34.30	75.6 ± 32.6^*^	0.66 (0.33–0.98)
Left-side bridge test	59.6 ± 32.2	70.21 ± 28.5^*^	0.43 (0.13–0.74)
Trunk flexor endurance test	80.7 ± 48.9	122.8 ± 65.8^*^	0.82 (0.51–1.13)
Biering-Sørensen test	79.2 ± 40.5	97.2 ± 35.7^*^	0.53 (0.29–0.77)
ODI 0–100 (>MDC)	31.6 ± 12.3	18.8 ± 13.9^*^ (34)	2.85 (3.15–2.55)
ODI: section 1—Pain intensity (0–5 scale)	2.1 ± 0.8	1.2 ± 1.0^*^	–

Standardized response mean (SRM) with 95% confidence interval (CI).

ODI, Modified Oswestry disability index; >MDC, number of participants who reported a change of ODI score superior to minimal detectable change (17/100) between baseline and follow-up. Significant change between baseline and the 6-week follow-up. *p < 0.001.

**Table 3:** B Number of participants function of the GRC score at the 6-week follow-up.

	GRC score								
	0	1	2	3	4	5	6	7	Total
Number of participants	9	5	6	9	12	16	22	5	84
	—————————— 75 ————————								
	——– 55 ——————								

### 3.2. Responsiveness

#### 3.2.1. Standardized response mean

SRMs were small for the left-side bridge test (0.43, 95% CI: 0.13–0.74), moderate for the right-side bridge test (0.66, 95% CI: 0.33–0.98), moderate for the Biering-Sørensen test (0.53, 95% CI: 0.29–0.77), and large for the trunk flexor endurance test (0.82, 95% CI: 0.51–1.13) and the ODI (2.85, 95% CI: 3.15–2.55) (Table 3).

#### 3.2.2. Minimal clinically important difference

The AUC of the ROC curves for each endurance test ranged from 0.57 to 0.67. Table 4 presents a selection of cut-off values for each test with priority being given to specificity/sensitivity or representing the best compromise between the two where appropriate. The ROC curves are reported in Supplementary material S2.

**Table 4:** Minimum clinically important difference estimated from receiver-operating characteristic curve analysis.

	AUC	Cut-off (s)	Specificity	Sensitivity
Right-side bridge test	0.58	15	0.54	0.60
		10	0.43	0.67
		5	0.39	0.78
Left-side bridge test	0.57	15	0.67	0.52
		10	0.52	0.61
		5	0.41	0.72
Trunk flexor endurance test	0.66	35	0.59	0.58
		25	0.59	0.65
		15	0.55	0.85
Biering-Sørensen test	0.67	15	0.64	0.57
		10	0.57	0.67
		5	0.54	0.74

Area under the curve (AUC), specificity and sensitivity for different cut-off values (s) for each of the endurance trunk tests.

AUC. area under the curve.

### 3.3. Longitudinal validity

No significant correlations were found between changes in the trunk muscle endurance tests and the change in ODI score (*r* < 0.15; all *P* > 0.05).

## 4. Discussion

The main purpose of our study was to assess the responsiveness of three trunk muscle endurance tests in military patients with LBP. Our results suggest a low to moderate ability of the side bridges and Biering-Sørensen tests to detect overall improvement in patients with LBP after a 6-week training program as shown by the SRMs. By contrast, the trunk flexor endurance test was highly sensitive to detect overall improvement in these patients as shown by large SRM. Moreover, we attempted to identify the MCIDs, that represent the minimal improvement in holding time needed at the completion of an intervention to produce a meaningful overall improvement, as perceived by patients. Our results showed a weak ability of trunk muscle endurance tests to discriminate between participants who were moderately to greatly improved from those who were stable to slightly improved as shown by the AUC lower than 0.70 for all tests.

According to our results, it is not possible, from the change in holding times, to discriminate between participants who were moderately to greatly improved from those who were stable to slightly improved at the completion of the 6-week training program. Moreover, the increases in holding time for each trunk muscle endurance test at the completion of the 6-week training program were statistically significant but below the minimal detectable change (MDC 95%: 29–138 s) reported in the literature (14, 15).These data may explain the low responsiveness of trunk muscle endurance tests in patients with LBP. Indeed, even if trunk muscles endurance tests are valid to assess trunk endurance muscle in patient with LBP (10), the performance do not rely solely on trunk muscles. Lower and upper limb muscles are involved in the tests and sensations of pain or discomfort in the back, or in the limbs can interfere with the holding duration. These factors taken all together are likely to influence the performance during trunk muscle endurance test and brought some individual to end the test before true trunk muscle fatigue. Indeed, 12.5% of the endurance tests performed in this study were stopped because of unbearable pain. Secondary analyses showed that baseline characteristics of participants who ended the test due to pain were not different than those of the other participants. However, in the cases of unbearable pain, true muscle fatigue was not reached thus we decided to exclude these results of the analyses. Clinicians should therefore be cautious when interpretating changes occurring during rehabilitation if pain is experienced during testing.

Our rehabilitation protocol included endurance, strength, coordination and stabilization exercises as recommended in the literature for patients with LBP (4, 6). These components are likely to improve muscle performance in order to increase core stability and load tolerance. However, we did not find any correlation between improvement in ODI and trunk endurance muscle performance when isolated. Similarly, Steele et al. (30) did not report any meaningful correlation between changes in isolated lumbar extension torque and changes in both pain intensity and disability in patients with LBP after resistance training programs. Their training programs included one or two sessions per week (for 12 weeks) of 8 to 20 repetitions of isolated lumbar extension with loads ranging 20%–80% of maximum voluntary contraction. From these results, it seems that the magnitude of improvement of muscle performance after a rehabilitation program for LBP, in terms of endurance and strength, is not likely to be related to enhancement in terms of overall improvement, pain and self-reported function.

Our results do not support the use of trunk muscle endurance tests to measure functional improvement. We may suggest that the ability to perform a complex task battery (according to patient needs) with proper core stability/movement pattern may be more appropriate to determine functional improvement. However, from our results and from the literature, we can also suggest that the improvement in overall condition, pain and disability observed in participants after completion of a training program may not be directly or solely attributable to changes in the musculoskeletal system (31, 32). It seems that improvement of the overall condition could be explained by other factors than the improvement of muscle endurance. Some hypothesis of mechanism of action of exercise such as load tolerance, self-pain efficacy and fear-avoidance reduction for example, could explain improvement following exercise programs and contribute to the improvement of both pain and function in patients with LBP (33). Therefore, self-reported questionnaires are still the gold standard to monitor improvement in terms of pain and function in military patients with LBP.

### 4.1. Strength and limitations

This study is the first to investigate the responsiveness of trunk muscle endurance tests in patients with LBP. Our results are clinically meaningful as we determined that improvement should not be based on holding time change assessment during trunk muscle endurance tests. The population recruited for this study was military members with LBP and most were men. Among them, less physically active participants (e.g., military members working in offices) were also recruited. Therefore, we think that our results may be generalized to civilian middle-aged men with LBP. Finally, knowledge of baseline performance by the evaluating physiotherapists and participants could have been a potential source of bias. However, baseline performance was not reported on the data collection sheet at final follow-up and it is very unlikely that the evaluating physiotherapists and participants remembered the results of the three tests performed 6 weeks earlier.

Trunk muscle endurance tests were performed in the same order for all the participants. Therefore, we cannot exclude a potential ordering effect. However, a 2-minute rest period was applied between each test to limit fatigue. The three muscle endurance tests used in this study were designed to assess trunk muscle endurance (11, 12). Without electromyographic recording we cannot ascertain that participant ended the test because of true trunk muscle fatigue. To limit this negative impact, we decided, in our analyses, to withdraw results from the data set when the test was ended due to an unbearable pain and not self-perceived fatigue. Finally, variability in holding time was very large within the group. However, our results are in agreement with previous results [non-symptomatic participants and healthy elite athletes (14, 15)], that also show high variability of trunk endurance muscle performance between participants (from 7 s to 300 s at baseline in our study).

## 5. Conclusion

Trunk muscle endurance tests demonstrated low to high ability to detect overall improvement after a 6-week training program in patients with LBP. Moreover, we were not able to determine an accurate threshold of holding time change in relation to a meaningful functional improvement and there was no association between trunk muscle endurance changes and functional improvement. Therefore, clinicians might use self-reported function measures rather than trunk muscle endurance tests to determine functional improvement in patients with LBP after completion of a training program.

## Data Availability

The raw data supporting the conclusions of this article will be made available by the authors, without undue reservation.
