# Supplementary material for: Is assessing trunk muscle endurance in military with sub-acute and chronic low back pain clinically meaningful?
Source: Front Sports Act Living. 2023 May 11;5:1173403. doi: 10.3389/fspor.2023.1173403 (PMC10211465; doi:10.3389/fspor.2023.1173403)
Supplement: Supplementary file 1 [file Presentation1.pdf]

frontiers

Supplementary Material

Is Assessing Trunk Muscle Endurance In Military With Sub-Acute And Chronic Low Back Pain Clinically Meaningful?

Pairot de Fontenay Benoit[1] (PT, PhD), Perron Marc[3] (PT, MSc), Gendron Chantale[c3,4] (PT, BSc), Langevin Pierre[3,5] (PT, MClSc), Roy Jean-Sébastien[2,3] (PT, PhD)

*** Correspondence:** Benoit Pairot de Fontenay: benoit.pdf@gmx.com

ILLUSTRATIONS OF THE ENDURANCE TEST'S POSITIONS

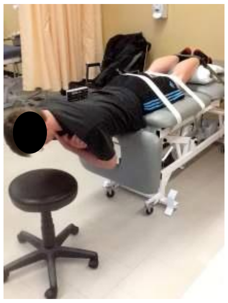

Biering-Sørensen test

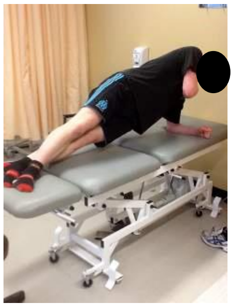

Side bridge test

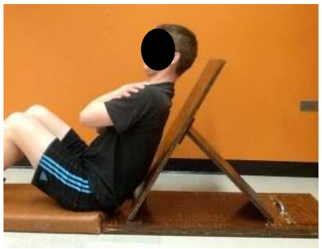

Trunk flexors endurance test

DESCRIPTION OF TESTING PROCEDURES

**Biering-Sørensen test**	Prone lying with the upper body off the table. The pelvis and lower limbs secured to the table with straps. Arms crossed on the chest. The participant lifts the trunk horizontally and keeps the position for as long as possible. The test ends when the participant is no longer able to maintain 10° above the horizontal despite verbal cues given by the assessor.
**Side bridge test**	Side lying, resting on the elbow. The opposite upper limb crosses the chest with the hand resting on the opposite shoulder. The participant lifts the pelvis and maintains the position as long as possible. The test ends when the participant is no longer able to keep the body alignment despite verbal cues given by the assessor.
**Trunk flexors endurance test**	Sitting with hips and knees at 90°, trunk supported on a backrest inclined at 30° from the vertical. Arms crossed on the chest. The assessor lowers the backrest 10° whilst the participant keeps the position for as long as possible. The test ends when the participant is no longer able to keep the position and makes contact with the backrests.

ILLUSTRATIONS OF THE EXERCISES INCLUDED IN THE MULTI-STATION PROGRAM

STATION 1: HIP STRENGTHENING AND CONTROL

1.1 Cat/Camel Back (1 X 8-10 repetitions)

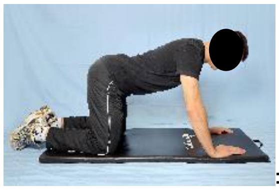
⇒
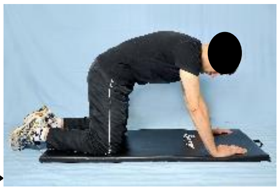

1.1 (A) **1.1 (B)**

1.2 Hip Flexion (2 X 5 repetitions)

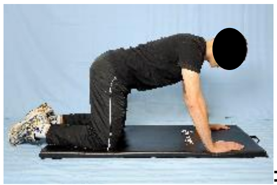
⇒
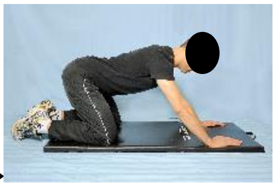

1.2 (A) **1.2 (B)**

1.3 Pivot (2 X 5 repetitions)

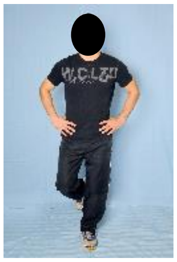
⇒
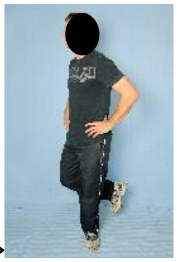
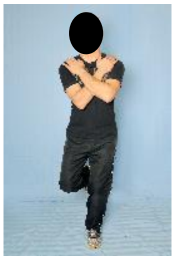
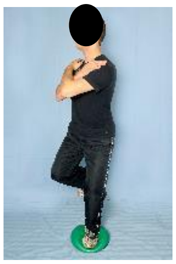
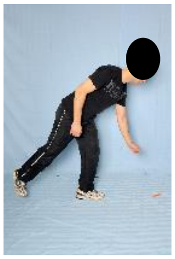
⇒
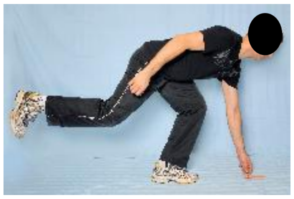

1.3.1 (A) **1.3.1 (B)** **1.3.2** **1.3.3** **1.3.4 (A)** **1.3.4 (B)**

1.4 Sit to Stand Transfer (2 X 5 repetitions)

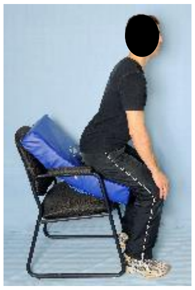
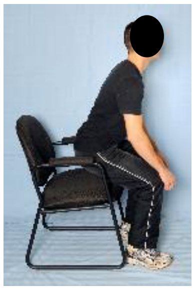
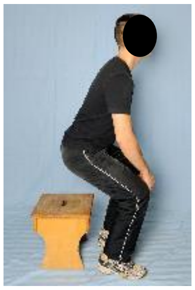

1.4.1 **1.4.2** **1.4.3**

1.5 ″ Japanese Stick ″ (2 X 30 seconds)

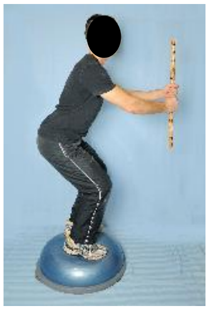
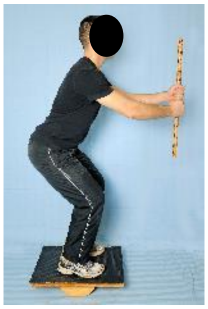
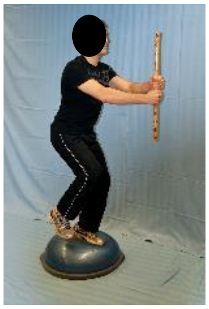
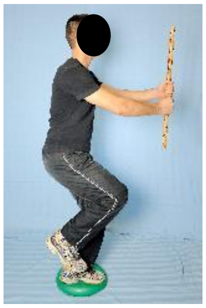
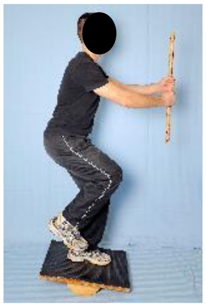

1.5.1 **1.5.2** **1.5.3** **1.5.4** **1.5.5**

1.6 Balance on a stool (2 X 30 sec)

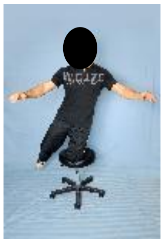

1.6.1

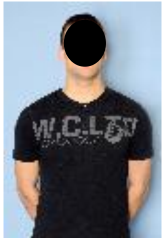

1.6.2

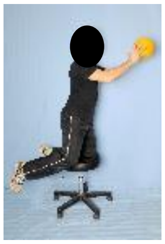

1.6.3

1.7 Targets on the wall (2X 30 sec)

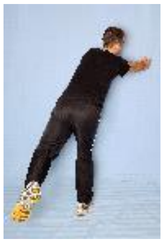

1.7.1

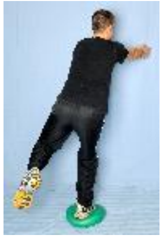

1.7.2

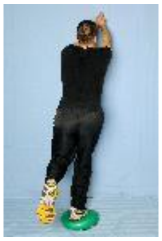

1.7.3

1.8 Hip Strengthening (hold 10 seconds, 2 X 10 repetitions)

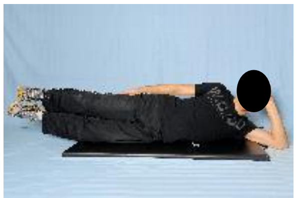

1.8.1

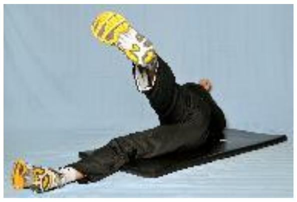

1.8.2

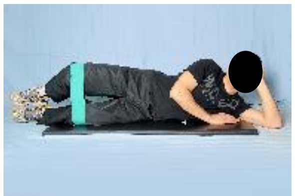

1.8.3

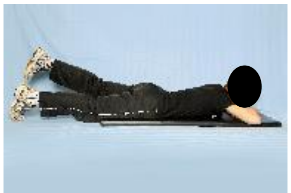

1.8.4

Station 2: THE SQUAT AND ITS VARIANTS

2.1 Half-squat (hold 20-30 seconds, 3 sets)

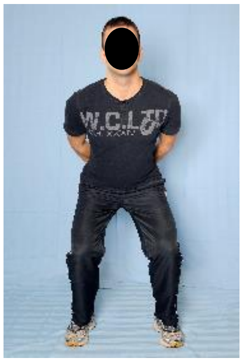

2.1.1

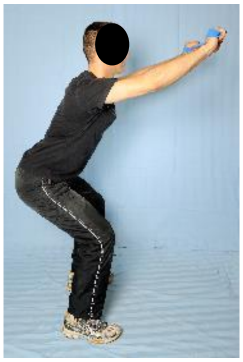

2.1.2

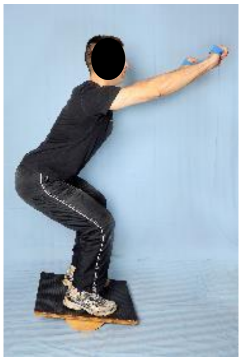

2.1.3

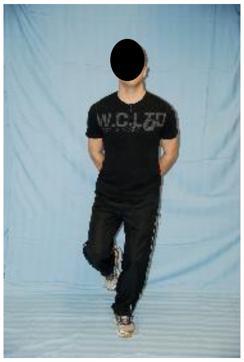

2.1.4

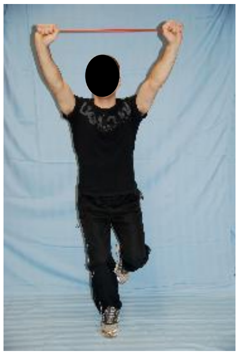 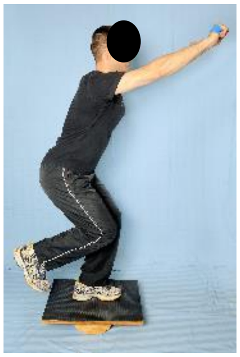 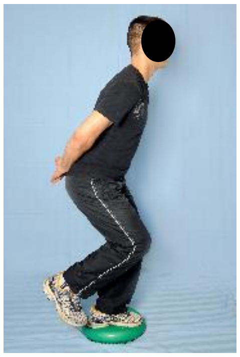

2.1.5 2.1.6 2.1.7

2.2 The Star (2 X 30 seconds)

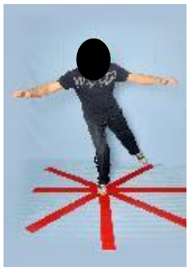

2.2.1

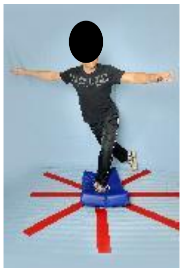

2.2.2

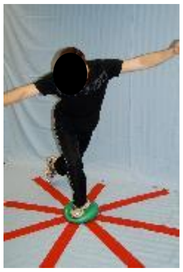

2.2.3

2.3 Lunge (2 X 10 repetitions)

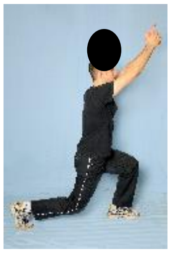

2.3.1

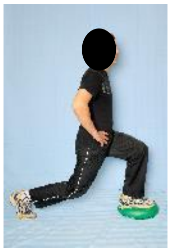

2.3.2

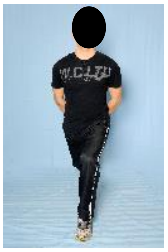

2.3.3

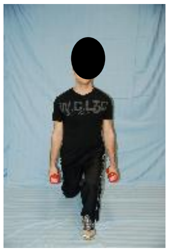

2.3.4

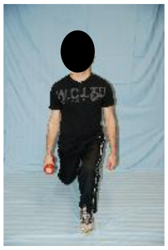

2.3.5

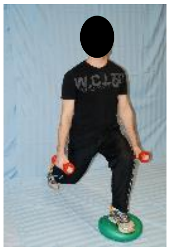

2.3.6

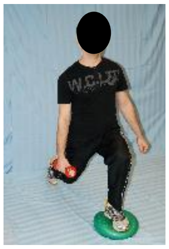

2.3.7

2.4 Shuttle (2 X 8-12 repetitions)

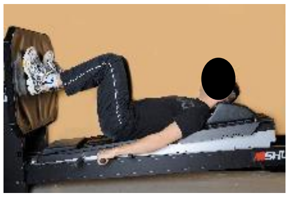

2.4.1 (A)

⇒

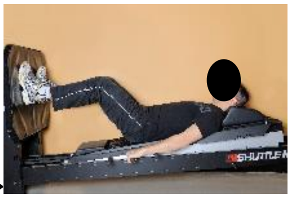

2.4.1 (B)

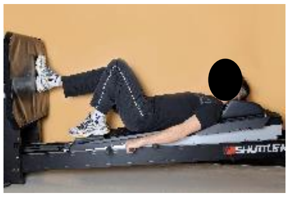

2.4.2

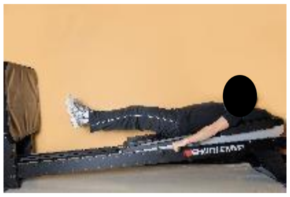

2.4.3

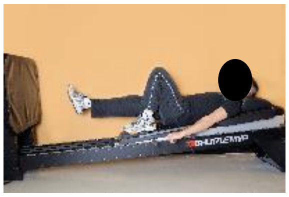

2.4.4

2.5 Jumps (2 X 10 repetitions)

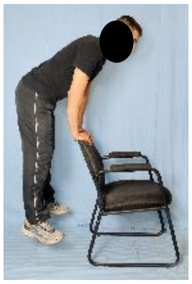
2.5.1

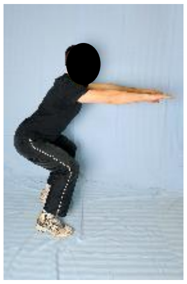
2.5.2

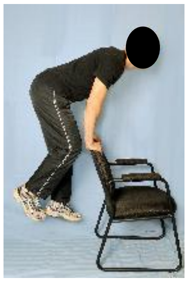
2.5.3

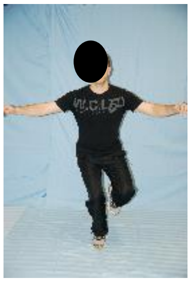
2.5.4

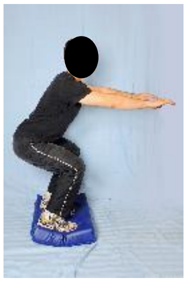
2.5.5

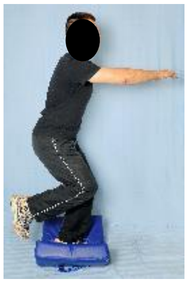
2.5.6

Station 3: ELASTIC BANDS AND THE BODYBLADE

3.1 Static position (hold 30 seconds, 2 sets on each side)

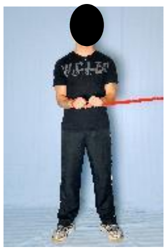
3.1.1

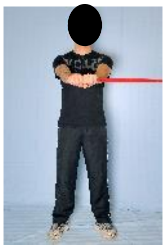
3.1.2

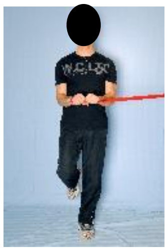
3.1.3

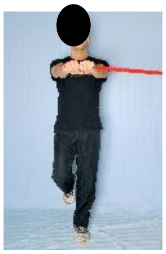
3.1.4

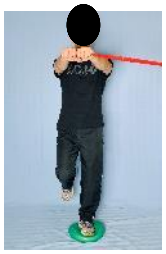
3.1.5

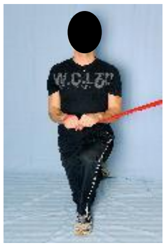
3.1.6

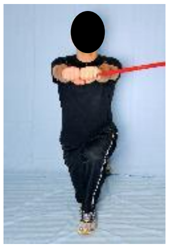
3.1.7

3.2 Dynamic movement of the upper limbs (2 X 10 repetitions on each side)

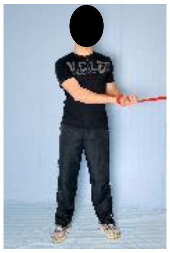
3.2.1 (A) ⇒

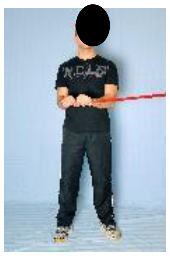
3.2.1 (B) ⇒

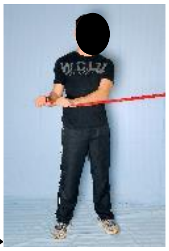
3.2.1 (C)

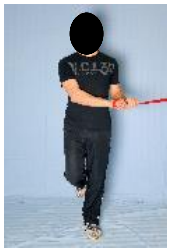
3.2.2 (A) ⇒

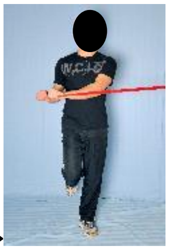
3.2.2 (B)

3.3 Dynamic rotation of the trunk (2 X 10 repetitions on each side)

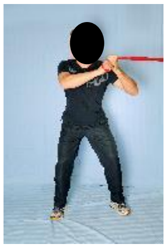 ⇒ 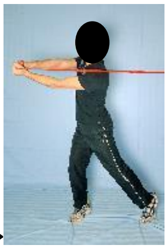 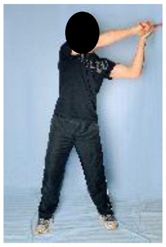 ⇒ 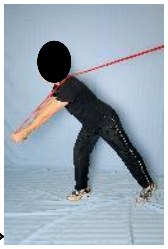 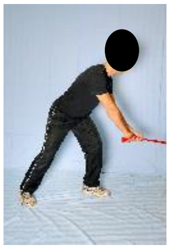 ⇒ 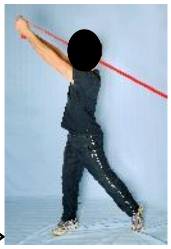

3.3.1 (A) 3.3.1 (B) 3.3.2 (A) 3.3.2 (B) 3.3.3 (A) 3.3.3 (B)

3.4 Bodyblade (3 X 20 seconds)

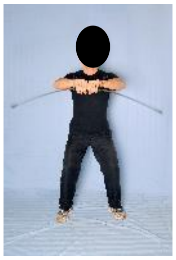 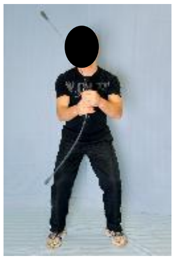 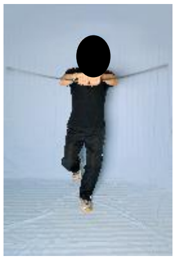 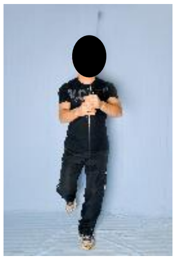

3.4.1 3.4.2 3.4.3 3.4.4

STATION 4: ABDOMINAL PLANKS AND THEIR VARIANTS

4.1 Push up position (3 X 20-30 seconds)

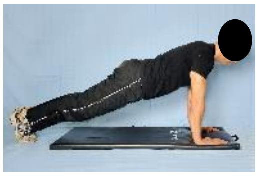 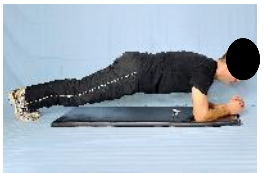 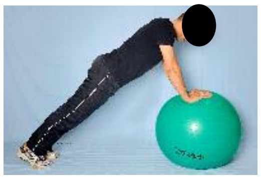 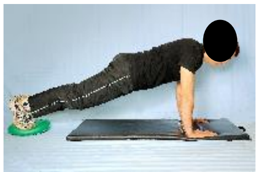

4.1.1 4.1.2 4.1.3 4.1.4

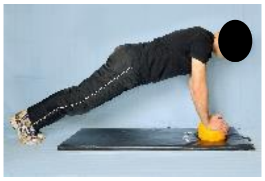 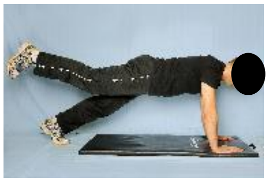 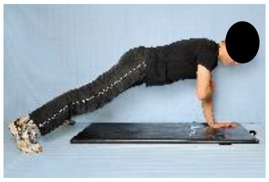 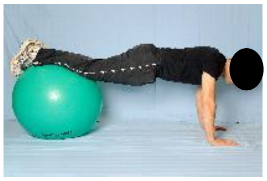

4.1.5 4.1.6 4.1.7 4.1.8

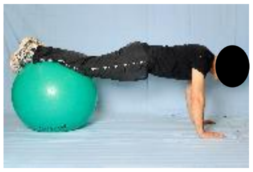 ⇒ 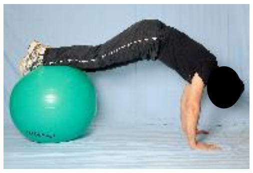 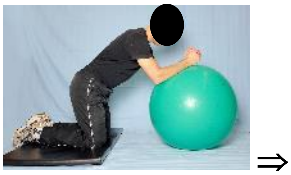 ⇒ 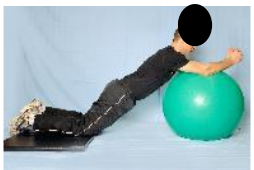

4.1.9 (A) 4.1.9 (B) 4.1.10 (A) 4.1.10 (B)

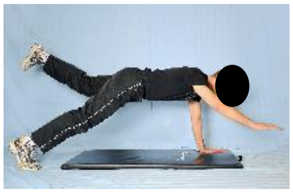

4.1.11

4.2 Pivot in the Push up position (2 X 20-30 seconds, alternate sides)

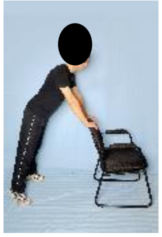 ⇒ 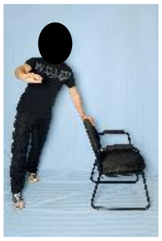 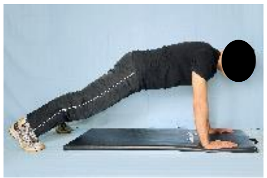 ⇒ 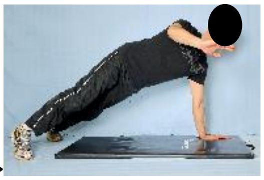 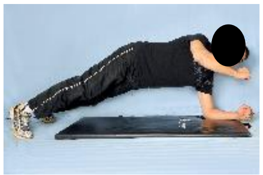

4.2.1 (A) 4.2.1 (B) 4.2.2 (A) 4.2.2 (B) 4.2.3

4.3 Sprint start from prone position (2 X 8-10 repetitions)

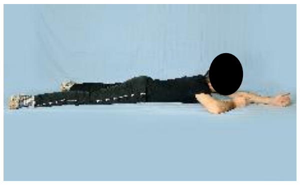 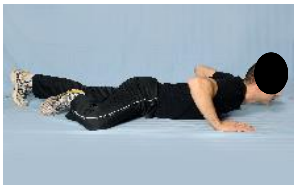 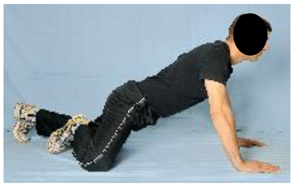 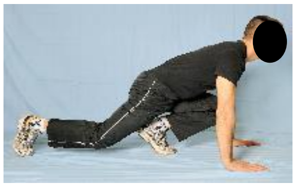

4.3 (A) 4.3 (B) 4.3 (C) 4.3 (D)

STATION 5: ABDOMINAL STRENGTHENING

5.1 « Curl up » (2 X 10 repetitions, hold position for 8 seconds)

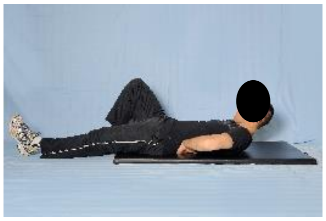
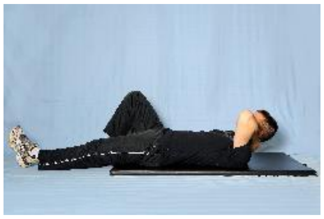
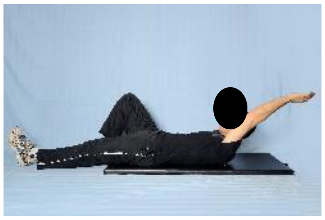

5.1.1 **5.1.2** **5.1.3**

5.2 Lateral plank (2 X 10 repetitions, hold position for 8 seconds)

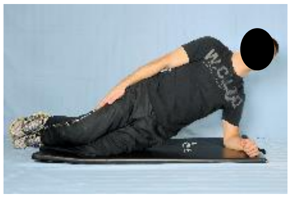
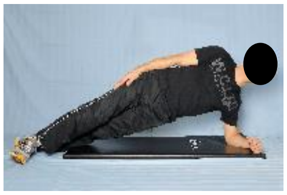
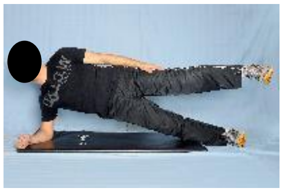
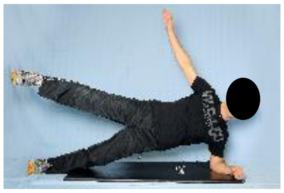

5.2.1 **5.2.2** **5.2.3** **5.2.4**

5.3 « Curl up » on a Swiss Ball (3 X 20-30 seconds)

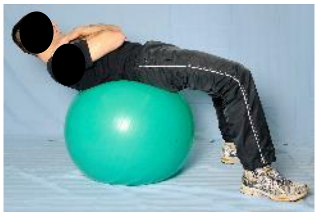
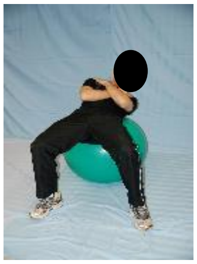
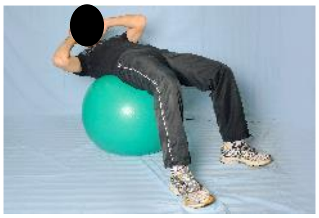

5.3.1 **5.3.2** **5.3.3**

5.4 Catching a medicine ball (3 X 20 seconds)

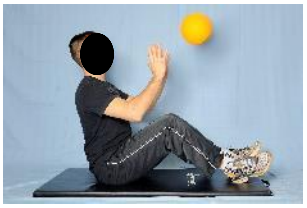

5.4.1

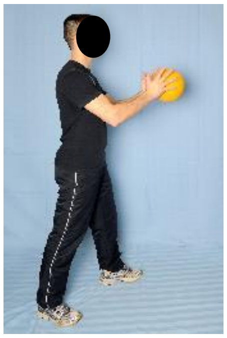

5.4.2

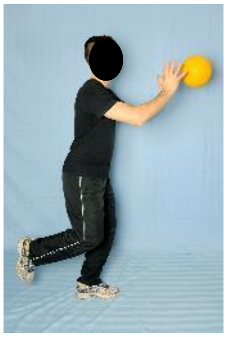

5.4.3

STATION 6: BACK EXTENSOR STRENGTHENING

6.1 « Bird Dog » (2 X 10 repetitions, hold position for 8 seconds)

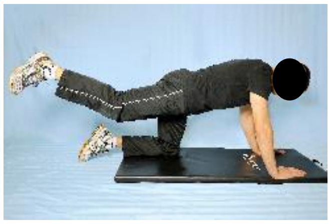

6.1.1

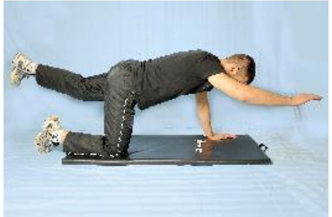

6.1.2

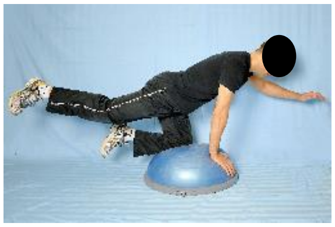

6.1.3

6.2 Strengthening on a Swiss Ball (2 X10 repetitions, hold position for 8 seconds)

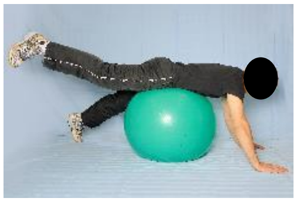

6.2.1

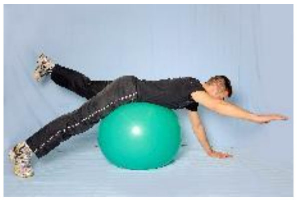

6.2.2

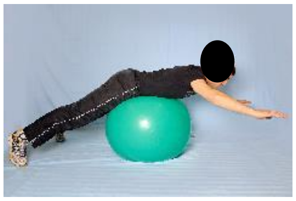

6.2.3

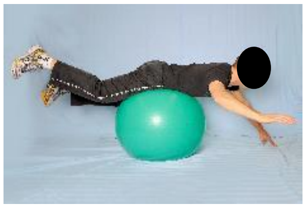

6.2.4

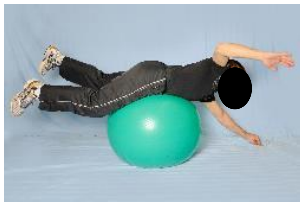

6.2.5

6.3 Lower limb extension (2 X 10 repetitions, hold position for 8 seconds)

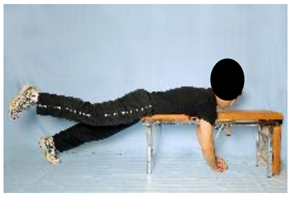

6.3.1

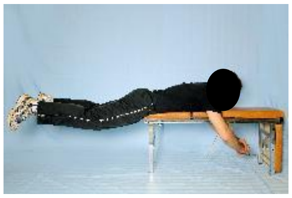

6.3.2

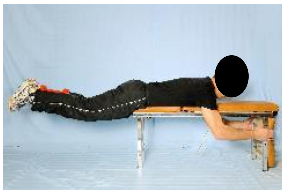

6.3.3

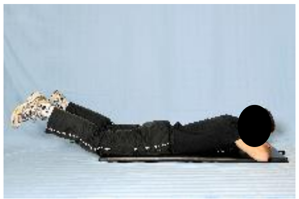

6.3.4

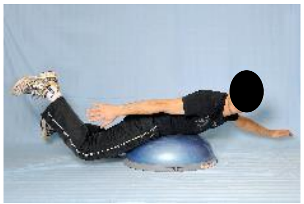

6.3.5

6.4 Bridge (2 X 10 repetitions, hold position for 8 seconds)

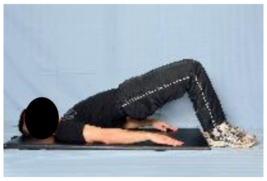

6.4.1

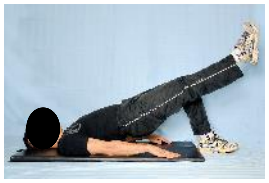

6.4.2

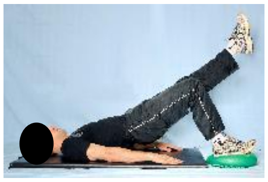

6.4.3

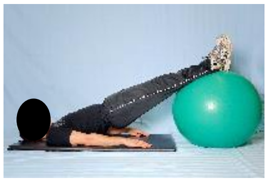

6.4.4

6.5 Roman Chair (2-3 X hold position for one minute)

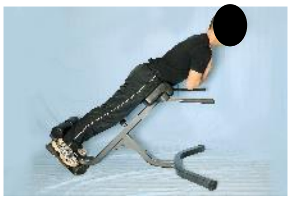

6.5.1

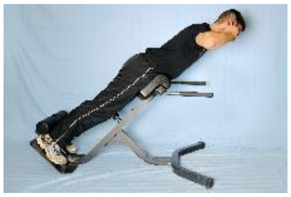

6.5.2

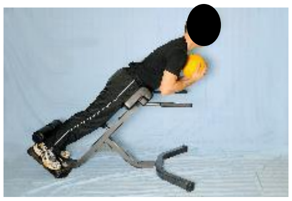

6.5.3

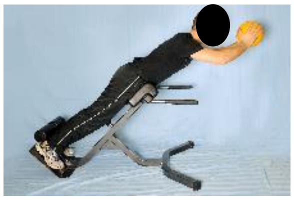

6.5.4

STATION 7: LIFTING TECHNIQUES

7.1 Deadlift (2-3 X 8-10 repetitions)

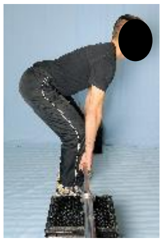

7.1.1 (A)

⇒

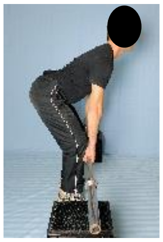

7.1.1 (B)

⇒

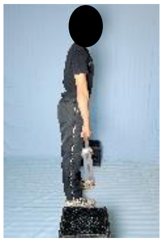

7.1.1 (C)

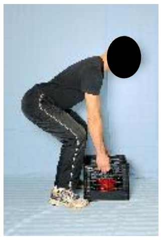

7.1.2 (A)

⇒

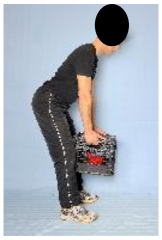

7.1.2 (B)

⇒

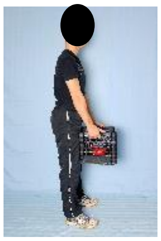

7.1.2 (C)

7.2 Weight transfers and walking with a load (2 X 5 repetitions)

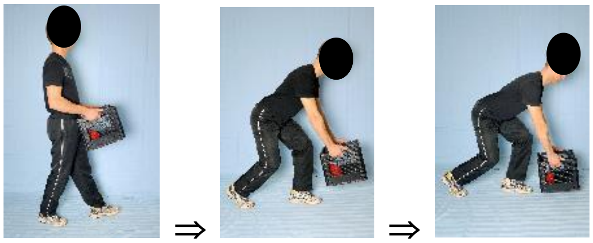

7.2.1 (A) 7.2.1 (B) 7.2.1 (C)

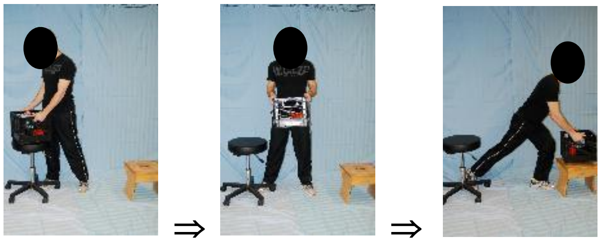

7.2.2 (A) 7.2.2 (B) 7.2.2 (C)

7.3 Unilateral lift (2 X 10 repetitions)

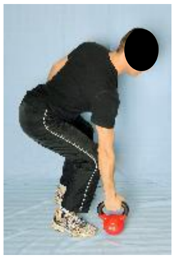 ⇒ 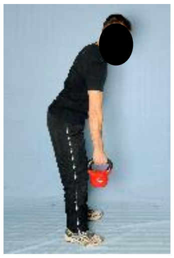 ⇒ 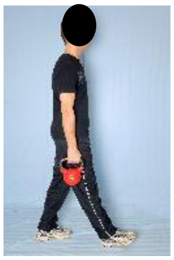 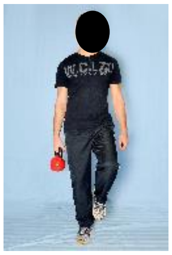

7.3.1 (A) 7.3.1 (B) 7.3.1 (C) 7.3.2

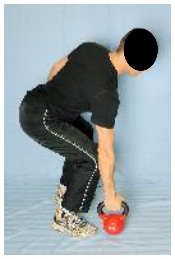 ⇒ 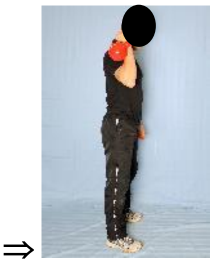 ⇒ 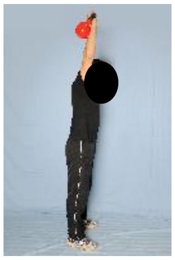 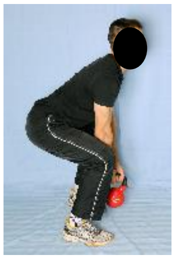 ⇒ 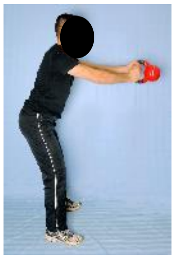 ⇒ 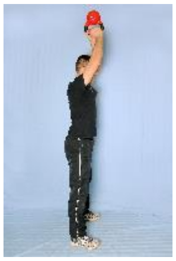

7.3.2 (A) 7.3.2 (B) 7.3.2 (C) 7.3.3 (A) 7.3.3 (B) 7.3.3 (C)

7.4 Lifting a Rucksack (1 X 5 repetitions)

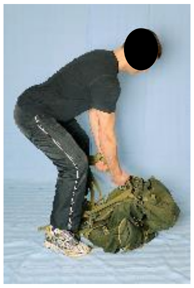 ⇒ 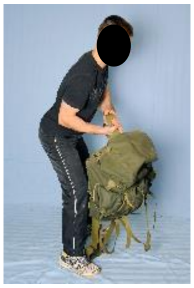 ⇒ 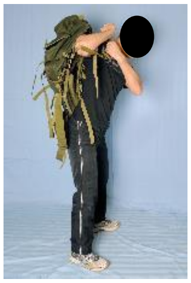

7.4.1 (A) 7.4.1 (B) 7.4.1 (C)

7.5 Casualty drag (2 X 5 repetitions)

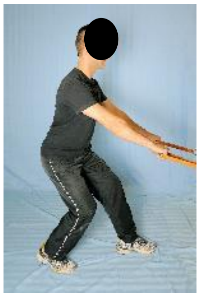 ⇒ 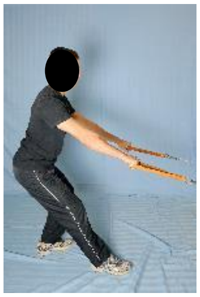

7.5.1 (A) **7.5 1 (B)**

Exercises parameters and selection

Basic principles applied for all exercises

- A natural lumbar lordosis should me maintained in all times, regardless of the weight or external load imposed on the body
- A large variety of exercises should be completed
- Focus should be kept on the quality rather than the quantity of movements

The selection of exercises and initial level of difficulty is determined according to 3 criteria:

1. The severity of the condition (pain at rest, disturbed sleep, level of limitation and restriction)
2. The most limited plane of motion: the prescribed exercises should be primarily carried out in the planes of motion that showed limited mobility or aberrant movements
3. The quality of exercises execution: maximal effort during exercises should not jeopardizing the quality of the movements.
